# A41 IDENTIFICATION OF MICROBIAL LYSOPHOSPHATIDYLCHOLINE (LPC) AND LYSOPHOSPHATIDIC ACID (LPA) PRODUCERS AND THEIR EFFECTS ON THE HOST

**DOI:** 10.1093/jcag/gwad061.041

**Published:** 2024-02-14

**Authors:** M Hall-Bruce, J Pujo, G De Palma, V Mohan, J Lu, S Collins, P Bercik

**Affiliations:** . Medical Science, McMaster University Faculty of Health Sciences, Hamilton, ON, Canada; . Gastroenterology, Farncombe Family Digestive Health Research Institute, Hamilton, ON, Canada; . Gastroenterology, Farncombe Family Digestive Health Research Institute, Hamilton, ON, Canada; . Medical Science, McMaster University Faculty of Health Sciences, Hamilton, ON, Canada; . Gastroenterology, Farncombe Family Digestive Health Research Institute, Hamilton, ON, Canada; . Gastroenterology, Farncombe Family Digestive Health Research Institute, Hamilton, ON, Canada; . Gastroenterology, Farncombe Family Digestive Health Research Institute, Hamilton, ON, Canada

## Abstract

**Background:**

Gut microbiota plays a key role in shaping our immune system, producing a myriad of molecules with neuro- and immunomodulatory properties. Many of these bioactive molecules are generated by bacterial metabolism of common dietary components. We have recently found that fecal samples of patients with chronic abdominal pain contain high levels of inflammatory phospholipids, lysophosphatidylcholine (LPC) and its active metabolite lysophosphatidic acid (LPA). Both phospholipids have been previously linked to genesis and maintenance of chronic neuropathic pain. In addition, they appear to have pro-inflammatory properties.

**Aims:**

To investigate whether gut bacteria can produce LPC and LPA from dietary precursor phosphatidylcholine (PC), and whether these phospholipids can induce inflammatory responses in the host.

**Methods:**

Fecal samples from patients with chronic abdominal pain were analyzed for microbial profiles using 16S rRNA (Illumina) analysis, and abundance of individual bacteria was correlated with fecal LPC and LPA levels as measured by ELISA. To confirm ability to produce these lipids, identified bacteria were incubated with either phosphatidylcholine (PC) or LPC (the immediate precursors of LPC and LPA, respectively) and LPC and LPA levels measured using liquid chromatography-mass spectrometry. To investigate pro-inflammatory effect of LPC and LPA, mouse splenocytes were incubated with LPC and/or LPA *in vitro*, and tested for cytokine production.

**Results:**

Higher abundance of several bacterial species was associated with higher fecal levels of LPC and LPA. Among them, a bacterium from family *Oscillospiraceae* was identified as the most likely producer of both LPA and LPC; its abundance also correlated with high pain scores. Culturing and incubating this bacterium with PC and LPC resulted in a significant production of LPC and LPA. Additionally, LPA, but not LPC, resulted in increased levels of IL-6 production by splenocytes *in vitro* compared to the PBS only control. Interestingly, LPA induced IL-6 secretion was inhibited in the presence of LPC.

**Conclusions:**

We have identified several bacterial producers of LPC and LPA, among them a bacterium from family *Oscillospiraceae,* which was also associated with high abdominal pain scores. LPA, but not LPC, seems to induce proinflammatory responses in the host. Further experiments are needed to identify the specific mechanisms by which these bioactive phospholipids induce abdominal pain and immune responses in the host.

**Funding Agencies:**

CIHRFarncombe Family Digestive Health Research Institute

